# Automated real-time text messaging as a means for rapidly identifying acute stroke patients for clinical trials

**DOI:** 10.1186/1745-6215-15-304

**Published:** 2014-07-29

**Authors:** Kati Jegzentis, Tim Nowe, Peter Brunecker, Matthias Endres, Bernd Haferkorn, Christoph Ploner, Jens Steinbrink, Gerhard Jan Jungehulsing

**Affiliations:** Center for Stroke Research Berlin (CSB) and Department of Neurology, Charité - Universitätsmedizin Berlin, Charitéplatz 1, 10117 Berlin, Germany; Department of Neurology, Charité University Hospital Berlin, Charitéplatz 1, 10117 Berlin, Germany; Department of Neurology, Vivantes Hospital, Berlin, Vivantes Klinikum Neukölln, Rudower Str 48, 12351 Berlin, Germany; Department of Neurology, Jewish Hospital Berlin, Heinz-Galinski-Str. 1, 13347 Berlin, Germany

**Keywords:** Acute trials, CIS, IT-based, Notification tool, Patient identification, Recruitment, Selection criteria, Stroke

## Abstract

**Background:**

Recruiting stroke patients into acute treatment trials is challenging because of the urgency of clinical diagnosis, treatment, and trial inclusion. Automated alerts that identify emergency patients promptly may improve trial performance. The main purposes of this project were to develop an automated real-time text messaging system to immediately inform physicians of patients with suspected stroke and to test its feasibility in the emergency setting.

**Methods:**

An electronic standardized stroke algorithm (SSA) was implemented in the clinical information system (CIS) and linked to a remote data capture system. Within 10 minutes following the documentation and storage of basic information to CIS, a text message was triggered for patients with suspected stroke and sent to a dedicated trial physician. Each text message provided anonymized information on the exact department and unit, date and time of admission, age, sex, and National Institute of Health Stroke Scale (NIHSS) of the patient. All necessary information needed to generate a text message was already available – routine processes in the emergency department were not affected by the automated real-time text messaging system. The system was tested for three 4-week periods. Feasibility was analyzed based on the number of patients correctly identified by the SSA and the door-to-message time.

**Results:**

In total, 513 text messages were generated for patients with suspected stroke (median age 74 years (19–106); 50.3% female; median NIHSS 4 (0–41)), representing 96.6% of all cases. For 48.3% of these text messages, basic documentation was completed within less than 1 hour and a text message was sent within 60 minutes after patient admission.

**Conclusions:**

The system proved to be stable in generating text messages using IT-based CIS to identify acute stroke trial patients. The system operated on information which is documented routinely and did not result in a higher workload. Delays between patient admission and the text message were caused by delayed completion of basic documentation. To use the automated real-time text messaging system to immediately identify emergency patients suitable for acute stroke trials, further development needs to focus on eliminating delays in documentation for the SSA in the emergency department.

## Background

Effective therapy of acute stroke is limited to the first few hours after stroke onset. Upon hospital arrival, the diagnostic workflow is of highest priority and treatment needs to be initiated as soon as possible [1]. Acute treatment trials offer additional therapeutic options for stroke patients. For hospital staff, however, identifying the patients who meet eligibility criteria for acute stroke trials within minutes can be challenging [1, 2]. Even in active academic centers the number of eligible patients is limited [3, 4], and only one third of acute treatment trials recruit the targeted number of patients within the time originally planned [5]. Thus, improving the recruiting process is of supreme importance. Lack of time in the emergency setting is one of the obstacles for the identification of acute trial patients [6].

A dedicated trial team, including trial physicians and trial assistants, was formed to improve trial performance in our hospital. The team recruits about 500 patients per year for clinical trials. Tables 1, 2 and 3 provide detailed information on the relevant studies and their corresponding inclusion rates.

**Table 1:** **Number of studies conducted by the stroke trial team per year**

	2010	2011	2012	2013
Interventional trials	10	10	9	6
Observational trials	16	19	16	17
Registries	1	1	1	1

**Table 2:** **Number of patients recruited by the trial team per year**

	2010	2011	2012	2013
Interventional trials	69	45	239	263
Observational trials	1,043	1,213	500	464
Registries	40	53	27	17
**Total**	**1,152**	**1,311**	**766**	**744**

**Table 3:** **Number of patients recruited by the trial team classified by time of symptom onset**

	2010	2011	2012	2013
Acute (<9 h)	27	52	14	41
Subacute (<36 h)	191	156	112	65
Subacute (<72 h)	61	41	27	86
Other	873	1,062	613	552
**Total**	**1,152**	**1,311**	**766**	**744**

To ensure that the trial physician on duty is rapidly notified when a patient arrives in the emergency department (ED), we implemented an automated screening tool that transmits the relevant data via text messaging to a mobile phone. In contrast to other automated screening systems we wanted to minimize time delay because acute interventional stroke trials are often limited up to 6 hours [7–9] or 9 hours [10, 11] from symptom onset. Even for interventional stroke trials that include patients up to 24 hours [12] from symptom onset or when eligibility is based on imaging criteria [12, 13], an immediate notification is necessary to guarantee that patient treatment is initiated promptly.

Here, we report on the technical development and organizational feasibility of an automated stroke alerting tool for emergency stroke patients.

## Methods

An electronic standardized stroke algorithm (SSA) consisting of a standardized stroke documentation sheet (SSS) and a text message sent to the study physician’s mobile phone was implemented in 2010; Table 4 provides more information on the SSS. As soon as a patient is registered in the ED as a patient with suspected stroke, the stroke sheet is compiled by the clinical information system (CIS). The SSS has to be completely filled out before the ED registration process can be closed. It contains routinely documented information on socio-demographic data such as age, sex, clinical status, date, time, and department and unit of admission, as well as information on stroke severity by the National Institute of Health Stroke Scale (NIHSS). No additional information is needed.

**Figure 1:**
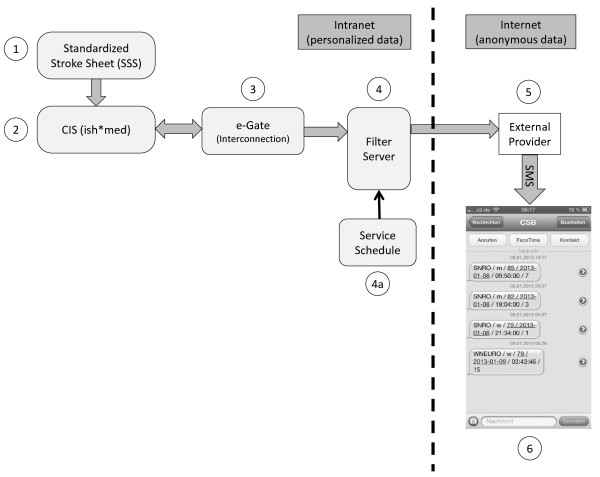
**Overview of the Standardized Stroke Algorithm (SSA) and the resulting text message.** (1) Physicians at the different EDs document routine clinical data in the electronic Standard Stroke Sheet (SSS) at the clinical workstations. (2) Data is saved and made available to interconnect by the clinical information system (CIS). (3) The CIS-server is polled by the e-Gate (interconnection gateway) every 10 minutes and data is retrieved from the CIS. (4) The filter server provides anonymous data and is controlled by a service desktop to adjust the filter settings. (5) The external provider sends the anonymized data to the (4a) pre-assigned recipients. (6) Screenshot of exemplary text messages received on mobile phone on January 8^th^, 2013, with information about hospital site and department as well as patient’s sex, age, date and time of admission, and NIHSS score. While the SSA operates on personalized patient data within the Charité intranet, no personal data is transmitted via internet.

The notification tool requires an IT-based ED document which is capable of automatically transmitting the patient information according to the HL7 standard. Text messages based on anonymized patient information are provided by a third-party telecommunications company. Once the SSS is completed, the data is filed in CIS (Figure 1). The CIS server is polled by the interconnection gateway every 10 minutes and retrieves data from the central medical information system. To comply with German regulations on data protection and data privacy, this personalized data is sent to an internal filter server which provides an anonymous dataset according to diagnostic criteria and sender. An event recognized as a probable stroke is immediately forwarded as a text message to the study physician on call and to the consultant neurologists via an external provider. The text message includes anonymized information on the hospital department and unit as well as the acute stroke patient’s sex, age (in years), date and time of admission, and NIHSS.

**Table 4:** **Information documented in the emergency department (ED)**

Demographic and organizational patient data	Information documented by admitting medical staff	Information documented by ED neurologist
- name	- current complaints	- general evaluation incl. neurological examination
- date of birth	- description of symptoms	- tentative diagnosis (ICD-10-Code)
- sex	- medical history	- indicated diagnostics (e.g., imaging) and results
- case ID	- risk factors (e.g., smoking behavior, hypertension, diabetes mellitus)	- medication administered
- treating department (e.g., emergency department)	- vital signs	- recommendations, for example admission to stroke unit
- date and time (d&t) of hospital admission	- concomitant medication	- NIHSS incl. single sub-points**
- d&t of first contact with physician	- leading symptoms (selection via radio button):	- information about stroke/TIA (selection via radio button):**
- means of patient transport to hospital (e.g., ambulance)	- no information	- no stroke or TIA
	- stomach ache	- stroke with alarm***
	- chest ache	- stroke without alarm
	- dyspnea	- TIA
	- stroke/transient ischemic attack (TIA)*	- d&t alarm**
	- headache	- d&t symptom onset *or* d&t last seen well**
	- none of these symptoms	- information about thrombolysis:**
		- yes/no if yes: d&t start of thrombolysis; if no: reason why not: (textfield)

*This selection triggers a new form for stroke-specific information, the standardized stroke sheet. Patients with suspected stroke/TIA will be examined by a neurologist.

**Standardized stroke sheet (SSS).

***Alarm will inform involved facilities (e.g., imaging unit, stroke unit, laboratory etc.) immediately. Information is sent by an automatic telephone chain.

The electronic standardized stroke algorithm was tested at the three campuses of the Charité University Hospital in Berlin. Altogether, about 2,000 patients with suspected stroke are admitted to the Charité every year. Table 5 provides details on each of the three campuses.

**Table 5:** **Information on campuses and admitted patients**

	CCM	CVK	CBF
Geographical location	Middle of Berlin (0 km)	About 3 km to the northwest	About 10 km to the south
Number of patients with suspected stroke per year	approx. 400	approx. 600	approx. 1,000

CCM: Charité Campus Mitte; CVK: Campus Virchow Klinikum; CBF: Campus Benjamin Franklin.

Technical and organizational feasibility of the SSA was tested by cohort analysis. For technical stability, we analyzed the number of text messages sent correctly by the system. For the organizational benefit of the system, door-to-message times and the number of correctly identified stroke-patients were examined. To monitor the completeness of the identified patients list, a manual screening of acute stroke patients was conducted by a trial physician. To balance cohort analysis, we compared datasets of consecutive patients during three months with empirically high (November), average (April), and low (June) stroke incidence rates [14]. To check inter-variability between cohorts, patients’ demographic data such as median age, sex, and median NIHSS, were analyzed.

## Results

To evaluate the feasibility of the electronic SSA, we analyzed exemplary text messaging alerts from three consecutive cohorts with a total of 513 alarms (November, n = 194; April, n = 183; July, n = 136). Median age in these cohorts was 74 years (Interquartile range (IQR), 63–81), 50.3% were female (n = 258), median NIHSS was 4 (IQR, 1–8). There were no significant differences between the three cohorts (Table 6) that were relevant for our analysis.

**Table 6:** **Demographics, NIHSS, and stroke subtype of patients identified by SSA (n = 513)**

	Total	November 2010	April 2011	June 2011	*P*
Text messages sent (n)	513	194	183	136	
Age, median (IQR)	73 (63–81)	74 (66–82)	73 (65–82)	72 (57–80)	0.05
Female sex,% (n)	50.3 (258)	55.2 (107)	51.4 (94)	41.9 (57)	0.06
NIHSS, median (IQR)	4 (1–8)	4 (1–9)	3 (1–8)	4 (1–7)	0.90
Time from admission to triggered text message (TTM), median in min (IQR)	62 (32–118)	61 (31–108)	64 (35–117)	66 (20–129)	0.39
**Tentative diagnosis at admission**					0.56
Ischemic stroke,% (n)	69.4 (356)	68.0 (132)	68.3 (125)	72.8 (99)	
Hemorrhages,% (n)	6.4 (33)	9.8 (19)	4.4 (8)	4.4 (6)	
TIA,% (n)	13.1 (67)	10.8 (21)	15.8 (29)	12.5 (17)	
Others,% (n)	11.1 (57)	11.3 (22)	11.5 (21)	10.3 (14)	

In the period of investigation, a total of 595 patients were diagnosed at admission with suspected stroke (ischemic or hemorrhage) in CIS. For 48 patients, the original diagnosis was refuted by the ED consultant, for 16 patients no standardized stroke sheet was compiled by CIS (patients were admitted to adjacent departments where SSA is not implemented). A text message to a mobile phone was correctly sent in 513 (96.6%) of the remaining 531 patients with suspected stroke. For 15 patients, no text message was generated because of hospital-wide technical modifications in CIS that affected the filter settings. For 3 patients, no text message was triggered.

Median time from admission to text-messaging was 62 minutes (IQR, 32–118) with 48.3% (n = 239) dispatched messages in less than 60 min and 27.3% (n = 135) within 60 to 120 minutes.

## Discussion

We demonstrate that a SSA that includes an automated text message to a mobile phone for acute stroke patients is technically feasible, and that the system maintains its stability. Only on two consecutive days were text messages (15 = 3.4%) missed as a result of technical modifications in CIS. As these adaptions were not made known to the users beforehand, no adjustments had been carried out in advance on our filter settings. Immediately after notification of CIS changes, filter settings were modified. No text messages were missed after these modifications.

In contrast to previous investigations [15–17], our approach was to focus on the special requirements for acute trials, which mandate start of treatment within a very limited time window: within 6 or 9 hours of symptom onset. To comply with selection criteria, patients must be screened immediately on arrival in hospital. In addition, relevant information, e.g., NIHSS, is essential for reaching a decision regarding a patient’s trial eligibility.

The SSA was implemented in the screening process of the neurological department and was designed as a technical interface to achieve rapid interaction between the physician on-call – who is responsible for routine diagnostic and treatment – and a specialized trial team physician. The SSA was based on information routinely documented in CIS and thus did not entail a higher workload for the treating physician. We think that this is of utmost relevance for the acceptance and feasibility of our screening tool in the emergency setting.

In contrast to other approaches, where selection criteria had to be defined beforehand to identify patients for specific trials [18], our method aimed at a more general technique: every single patient with suspected stroke was identified by the system and a text message with the most relevant screening data was generated. Text messages were sent to the trial physician on call, who was responsible for the screening and recruitment process for a multitude of stroke trials. Based on the information provided – patient sex and age, date and time of admission, and NIHSS – the trial physician pre-selected the most likely eligible patients for a variety of stroke trials. Naturally, a detailed screening process followed: the screening information supported the trial physician in deciding which patient to see first, i.e., in which case time was most pressing.

As in previous approaches, some patients were identified by the automated screening system which had not been identified in a manual search [16–18]. However, a further analysis showed that these additional patients were stroke mimics or were not admitted to our department, and were thus not eligible for acute stroke trials.

An advantage of our reporting tool was that absolutely all stroke patients were reported to the trial physician. Even patients who failed to meet inclusion criteria at admission were recorded and surveyed later to catch changes in medical condition, e.g., in NIHSS, and later trial eligibility.

As our alerting system did not depend on specific selection criteria, it was easily expanded for new trials or after modification of selection criteria in ongoing trials; no adaptions to the system were necessary. This was especially advantageous for repeated changes in selection criteria or for high turnover of trials.

The main focus of our approach was not only on capturing absolutely all patients but also generating the most rapid alert possible. However, the time from admission to text-messaging still exceeds 60 minutes in the majority of patients, which is rather long. In the emergency setting, documentation had to take second place to acute diagnosis and treatment. This led to delays in documentation and is in fact the major obstacle to using the automated screening tool for acute stroke intervention trials; for trials with a very limited time window, delays exceeding 60 minutes post-hospital admission can reduce trial eligibility. Nevertheless, in our case, delays were due not to technical flaws but rather to suboptimal organization. We aim to solve this problem with better training for the physicians in ED. Nonetheless, we have to take into account that delays in documentation will continue because therapy and treatment are more relevant for the patient than data documentation. In addition, future adaptions to the standardized stroke sheet will be mainly in the area of supplementary information (e.g., time of symptoms onset) for the screening process and the decision on trial eligibility.

A major benefit of the automated screening tool is its availability outside regular working hours. In our survey, 118 (23.0%) patients were admitted at weekends and a further 127 (24.8%) on working days between 6 pm and 6 am. We estimate that the majority of these 245 (47.8%) patients would not have been screened at all for trial eligibility without the automated notification tool.

Additionally, the notification tool might be of interest as an automated alerting system for patients with suspected stroke in standard care as the tool could inform all relevant services within minutes once an appropriate trigger is determined. The screening tool requires an IT-based ED file and HL7. As HL7 is highly available in most systems, the tool could be used at other hospitals as well. Costs of the notification system depend on the number of sent text messages and the specified charges.

### Limitations

We deliberately did not perform a comparison of total trial recruitment numbers with or without the notification tool, so no quantitative correlation for total recruiting numbers is provided.

## Conclusions

Our SSA including an automated text message to a mobile phone for acute stroke patients was technically stable and feasible. Data protection rules and privacy laws were obeyed. Standard procedures in the emergency setting were not affected. Nevertheless, documentation in the ED needs to be sped up to minimize delay between patient admission and text message or real-time screening of trial eligibility. An IT-based ED file and HL7 are required. As both are readily available in the clinical setting, the notification tool can be set up elsewhere. The benefits of the automated alerting tool are that its technical prerequisites are readily available and that it provides full documentation, can be utilized even outside working hours, is low in cost, and can be integrated into routine ED processes.

## Abbreviations

CIS: Clinical information system
CSB: Center for Stroke Research, Berlin
ED: Emergency department
IQR: Interquartile range
NIHSS: National Institute of Health Stroke Scale
SSA: Standardized stroke algorithm
SSS: Standardized stroke documentation sheet.
